# Strongyloidiasis Treatment Outcomes: A Prospective Study Using Serological and Molecular Methods

**DOI:** 10.3390/tropicalmed10040091

**Published:** 2025-04-01

**Authors:** Ana Lucas Dato, Philip Wikman-Jorgensen, José María Saugar Cruz, Elisa García-Vázquez, Jara Llenas-García

**Affiliations:** 1 Internal Medicine/Infectious Diseases Department, Vega Baja Hospital-Orihuela, 03314 Alicante, Spain; jarallenas@gmail.com; 2 Foundation for the Promotion of Health and Biomedical Research of the Valencia Region (FISABIO), 46020 Valencia, Spain; 3 Internal Medicine/Infectious Diseases Department, Elda General University Hospital, 03600 Alicante, Spain; 4 Department of Clinical Medicine, Miguel Hernandez University of Elche, 03202 Elche, Spain; 5 Laboratory of Reference and Research in Parasitology, Centro Nacional de Microbiología, Instituto de Salud Carlos III, 28222 Majadahonda, Spain; jmsaugar@isciii.es; 6 Biomedical Research Networking Center of Infectious Diseases (CIBERINFEC), Carlos III Institute, 28029 Madrid, Spain; 7 Infectious Diseases Unit, Hospital Virgen de la Arrixaca, 30120 Murcia, Spain; elisagarciavazquez@gmail.com; 8 Medicine Department, University of Murcia, 30003 Murcia, Spain

**Keywords:** strongyloidiasis, molecular diagnosis, follow-up, ivermectin, treatment

## Abstract

Strongyloidiasis, caused by the soil-transmitted helminth *Strongyloides stercoralis*, is estimated to infect around 600 million people worldwide. Ivermectin is the current first-line treatment. This prospective study evaluated long-term treatment response in patients with chronic strongyloidiasis. Conducted from 2019 to 2022 at Vega Baja Hospital in Alicante, Spain, this study enrolled 28 patients diagnosed with *S. stercoralis* infection. Patients received ivermectin at a dosage of 200 mcg/kg for one or two days and were followed for at least 12 months, with evaluations at 3, 6, 12, and 18 months post-treatment. Assessments included hemogram, IgE, *Strongyloides* serology, larvae culture and direct visualization and *Strongyloides* PCR in stool. Twenty-three patients completed at least 12 months of follow-up. Twenty-one patients (91.3%) achieved treatment response. Two patients (8.6%) experienced parasitological treatment failure, with detectable *Strongyloides stercoralis* DNA during follow-up. Ivermectin is highly effective in treating strongyloidiasis, with serology aiding in monitoring treatment efficacy. However, PCR detected an additional case of persistent infection, underscoring its complementary role.

## 1. Introduction

Strongyloidiasis is a widespread infection affecting an estimated 614 million people worldwide [1,2,3]. *Strongyloides stercoralis* is an intestinal nematode with a unique autoinfective cycle that allows it to persist in the host for decades, leading to chronic infections if left untreated. Many infections remain asymptomatic or present with mild gastrointestinal or dermatological symptoms. However, in immunosuppressed individuals, the parasite can cause hyperinfection syndrome (HIS) and disseminated strongyloidiasis (DS), conditions associated with high morbidity and mortality [4,5,6]. Diagnosing *S. stercoralis* remains difficult, and there is currently no universal gold standard for laboratory diagnosis [7,8]. Intermittent excretion of larvae in stool reduces the sensitivity of microscopic examination [9]. Serology has demonstrated high sensitivity, but its specificity can be suboptimal due to possible cross-reactivity with other helminth infections [10]. Moreover, sensitivity is decreased in immunosuppressed patients [11,12]. These limitations have led to the development of more advanced molecular techniques. Among these, polymerase chain reaction (PCR) assays have demonstrated high specificity and moderate sensitivity for detecting *S. stercoralis* DNA in stool samples, offering a potential alternative for diagnosis [13,14].

Ivermectin is currently considered the most effective therapeutic option [15]. However, achieving a parasitological cure is challenging. This is due to the parasite’s ability to evade immune responses and its potential for autoinfection, posing a continuous risk of disease dissemination. Some fatal cases of disseminated strongyloidiasis have been reported in patients who had undergone HIS treatment and were even initially considered cured [5]. In this sense, monitoring treatment response is another crucial aspect of managing strongyloidiasis. Traditional diagnostic methods, such as stool microscopy, have low sensitivity. In contrast, serological and molecular techniques, including real-time polymerase chain reaction (RT-PCR), have demonstrated greater accuracy in detecting persistent infections [13]. Despite advances in diagnostics and treatment, long-term follow-up is necessary to confirm cure and prevent relapse, especially in populations at risk of reactivation [8,16].

This study aims to evaluate the effectiveness of ivermectin therapy in the treatment of *S. stercoralis* infection, assess the role of serological and molecular tests in monitoring response to treatment, and analyze long-term follow-up outcomes in affected patients. As a secondary objective, we aimed to analyze whether there were differences in treatment response between autochthonous and imported cases.

## 2. Materials and Methods

### 2.1. Study Design

This observational prospective study enrolled patients diagnosed with strongyloidiasis at Vega Baja Hospital between April 2019 and September 2022. Vega Baja Hospital is in the Orihuela Health Department, Alicante, in southeastern Spain, serving a population of approximately 166,000 [17]. The region has a strong agricultural tradition, particularly in citrus and vegetable production, and is considered an endemic area for strongyloidiasis, with documented past transmission.

### 2.2. Inclusion Criteria

The inclusion criteria were (1) adult patients aged 18 years or older with a confirmed diagnosis of strongyloidiasis; (2) patients who have received at least one dose of ivermectin treatment in the past 12 months; and (3) patients who agreed to participate in and attend follow-up visits. Diagnosis was based on at least one of the following criteria: (i) positive real-time polymerase chain reaction (RT-PCR) for *Strongyloides* spp. in feces or sterile fluid (e.g., cerebrospinal fluid); (ii) visualization of larvae in stool culture, coproparasitological examination, or other specimens (e.g., sputum) in cases of hyperinfection syndrome; (iii) positive serology for *Strongyloides* spp.; or (iv) histological evidence of strongyloidiasis (visualization of larvae in tissue).

A case was considered autochthonous if the patient had resided in the Vega Baja region for more than 30 years and had no history of long-term travel to another *Strongyloides*-endemic area. Cases were defined as imported if the patients were migrants from or travelers to other endemic areas.

Patients were considered immunosuppressed and at risk for developing HIS if they presented with any of the following conditions at enrollment: (1) treatment with corticosteroids (≥20 mg/day prednisone equivalent) for at least 14 days; (2) treatment within the previous six months with cytotoxic drugs (e.g., chemotherapy, alkylating agents (e.g., cyclophosphamide, chlorambucil, melphalan), antimetabolites (e.g., methotrexate > 0.4 mg/kg/day, 6-mercaptopurine > 1.5 mg/kg/day, azathioprine > 3 mg/kg/day, 5-fluorouracil)), or biologics (e.g., anti-TNF, rituximab, or other cellular or humoral immunosuppressive drugs)); (3) bone marrow or solid organ transplantation; (4) active stage IV solid neoplasia or active hematological malignancy; (5) radiation therapy within the previous six months; (6) hypogammaglobulinemia; or (7) HIV infection with a CD4 lymphocyte count below 350 cells/mm^3^.

Eosinophilia was defined as a peripheral blood eosinophil count of 500 cells/mm^3^ or more. Elevated IgE was defined as a level of 100 IU/mL or more.

### 2.3. Study Procedures

Demographic and clinical data were collected, including age, sex, involvement in agricultural activities, access to sanitation and hygiene, history of parasitosis, and presence of gastrointestinal, respiratory, or cutaneous symptoms. Participants were screened for human immunodeficiency virus (HIV) and human T-lymphotropic virus 1 (HTLV-1) infections.

At baseline, blood and stool samples were collected from each patient for the following assessments: complete blood count (CBC), IgE measurement, *Strongyloides stercoralis* serology, coproparasitological examination, *Strongyloides stercoralis* culture, and DNA detection by real-time polymerase chain reaction (RT-PCR) from a fresh stool sample. All these tests, except the *Strongyloides* RT-PCR, are part of the routine baseline evaluation in our clinic for patients with strongyloidiasis.

### 2.4. Serological, Molecular, and Parasitological Studies

Serological testing was performed at the National Center for Microbiology (Instituto de Salud Carlos III, Majadahonda, Madrid, Spain) using a non-automated IVD-ELISA (DRG Instruments GmbH, Marburg, Germany) for the detection of IgG antibodies against crude filariform larval antigen. The positive and negative controls used are those provided by the commercial kit. We calculated the optical density (OD) index by dividing the OD value corresponding to each sample or control by the fixed cut-off value established by the commercial kit (0.2 OD units). In our practice, we consider index values between 1 and 1.1 (cut-off + 10%) indeterminate, and we define index values greater than 1.1 as positive.

Microscopic stool examination was performed using the Mini Parasep SF (Alcorfix, Apacor Limited, Berkshire, UK). *Strongyloides* culture was performed by seeding Mueller–Hinton agar, incubating at 28 °C, and observing for five days for characteristic sinuous tracks. Coproparasitological examination and fecal culture were performed at the microbiology laboratory of Vega Baja Hospital. Molecular detection of *Strongyloides stercoralis* DNA was performed using a qualitative real-time polymerase chain reaction (qPCR) assay. Stool samples for RT-PCR were stored at −20 °C until processing. PCR was also carried out at the Spanish National Centre for Microbiology (Instituto Nacional de Salud Carlos III; Majadahonda, Madrid, Spain), our clinical reference laboratory.

### 2.5. Patient Treatment

Patients were treated with ivermectin at a dosage of 200 mcg/kg for one or two days, at the discretion of the treating physician. The initial protocol recommended two doses of ivermectin. Subsequently, after the publication of the Strong Treat 1 to 4 study [7], a single dose of ivermectin became the preferred treatment, with a two-dose regimen used mainly for immunocompromised patients. Treatment was initiated empirically in cases where immunosuppression could not be deferred and in patients with suspected or confirmed hyperinfection syndrome, complicated strongyloidiasis, or disseminated strongyloidiasis.

### 2.6. Follow-Up Data

Patients were followed up to evaluate clinical response (presence of symptoms compatible with strongyloidiasis), hematological response, and parasitological response. Assessments were performed at 3, 6, 12, and 18 months post-treatment. At each visit, serum and stool samples were collected from every patient for the following assessments: complete blood count (CBC), IgE measurement, *Strongyloides* serology, coproparasitological examination, *Strongyloides* culture, and DNA detection by real-time polymerase chain reaction (RT-PCR) from a fresh stool sample. In our clinical practice, follow-up is carried out with CBC and *Strongyloides* serology, with *Strongyloides* culture and coproprasitological examination being added if they were positive at baseline. IgE, *Strongyloides* RT-PCR and systematic coproparasitological and *Strongyloides* culture were added for research purposes. A minimum follow-up period of 12 months was required to evaluate treatment outcomes.

Treatment response was defined as the clearance of *Strongyloides stercoralis* infection at 12 months, evidenced by a negative agar plate culture or PCR and negative or decreasing *Strongyloides* serology. A decrease in serological titer was defined as a twofold reduction in the OD index (ELISA) [7].

Partial treatment response was defined as a negative agar plate culture or PCR and decreasing *Strongyloides* serology, with the titer remaining above the cut-off for clearance.

Treatment failure was defined as a positive agar plate culture or PCR and/or increasing *Strongyloides* serology [18].

### 2.7. Statistical Analysis

For the descriptive analysis, categorical variables are presented as absolute and relative frequencies. The normality of continuous variables was assessed using the Kolmogorov–Smirnov test. Non-normally distributed continuous variables are described as medians and interquartile ranges (IQRs). In the univariable analysis, categorical variables were compared using Fisher’s exact test, and continuous variables were compared using the Mann–Whitney U test. A *p*-value of less than 0.05 was considered statistically significant.

### 2.8. Ethical Considerations

This study was conducted in accordance with the World Medical Association’s Declaration of Helsinki. National regulations regarding clinical research and data protection were always adhered to. This study was reviewed and approved by the Elche General University Hospital Research Ethics Committee (PI 14/2019). Written informed consent was obtained from all participants before enrollment in this study.

## 3. Results

**Figure 1:**
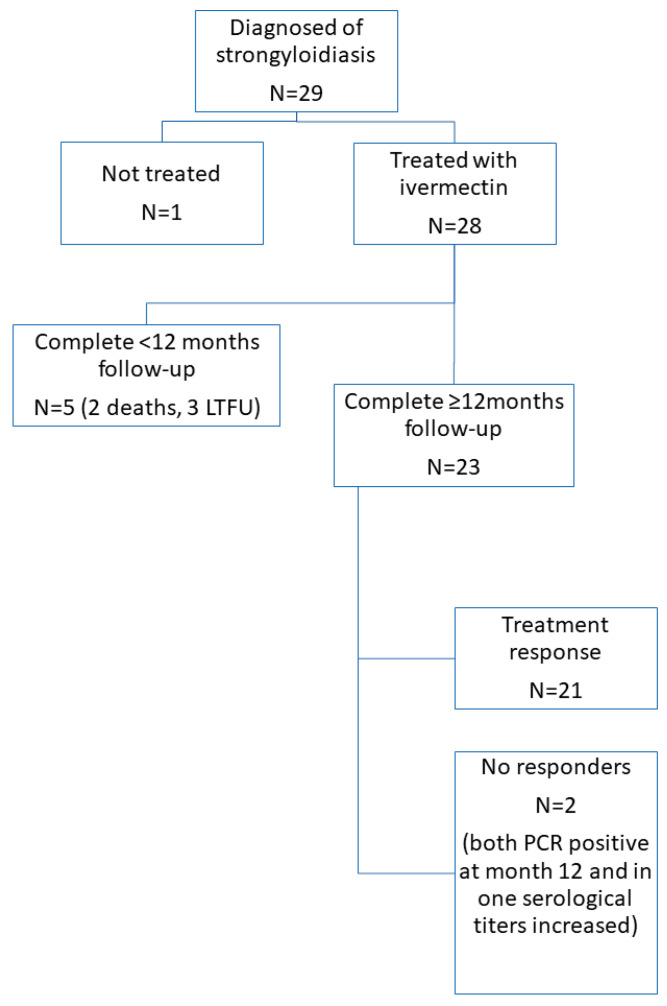
Study flow chart.

In total, 29 patients with strongyloidiasis were recruited from January 2019 to September 2022, of whom 28 received at least one dose of ivermectin and were finally included in this study (Figure 1).

There were 14 females (50%), and the median age at the time of diagnosis was 54.5 (IQR41.0–66.5) years. Twenty (71%) were classified as imported cases and 8 (29%) as autochthonous cases (Table 1). South America was the geographical area of origin in 18 (62%) patients, mainly coming from Bolivia *(n* = 13), Ecuador (*n* = 2), Colombia (*n* = 2), and Venezuela (*n* = 1). Only one patient came from North Africa (Morocco) and one from Central America (Honduras). The median time living in Spain was 14.8 years (12.7–17.4).

A high percentage of patients (22/28; 78.5%) had been involved in agricultural activities, with a median dedication to agriculture of 11 years (IQR 2–37 years), which was higher among autochthonous than imported ones (44 vs. 3 years; *p* = 0.02). Of those, 22 patients with involvement in agricultural activities, only 12 did so professionally (10 collectors and 2 vegetable packers).

Three patients had an immunosuppressant condition: one had a hematological neoplasia, and two were receiving immunosuppressive therapies (one with corticosteroids and the other with antimetabolite drugs). There were two patients with HIV with a CD4+ cell count > 350 cells/mm^3^.

At the time of diagnosis, eosinophilia was observed in 15/28 patients (53.57%) with a median baseline eosinophil count of 510 eosinophils/mm3 (IQR (205–792)) cells/mm^3^, and IgE was elevated in 9/23 patients (39.13%) with a median of 371 UI/mL (148–609).

Serological and parasitological results at baseline and at each subsequent visit are shown in Table 2. All three immunocompromised patients had a positive baseline serology, while baseline stool PCR was positive in 2/2, including one patient with a positive but very low serological titer (OD 1.11).

HTLV-I/II serology was negative in 19/19 cases.

All participants were treated—26 upon strongyloidiasis confirmation and 2 empirically. Seventeen were treated with ivermectin 200 mcg/kg/day in two doses, one week apart, while 11 received a single dose of ivermectin 200 mcg/kg. One patient also received albendazole 400 mg twice daily for 28 days for neurocysticercosis. None of them reported adverse effects.

Twenty-three patients (82.1%)—17 imported and 6 autochthonous patients—completed the minimum 12-month follow-up. Eight patients had their last visit at 12 months, and 15 patients had their follow-up after 18 months (Figure 1). The median follow-up was 14.4 months (IQR:8.0–21.3).

Among those 23 patients, 21 patients (91.3%) met the criterion for treatment response, and two patients (8.6%) exhibited parasitological treatment failure despite ivermectin therapy; both were imported cases. The first case involved an immunocompetent patient with no relevant medical history who received a single dose of ivermectin. Although this patient achieved serological seroreversion, real-time polymerase chain reaction (RT-PCR) remained persistently positive at both the 6-month and 12-month follow-up assessments.

The second case was an immunosuppressed patient undergoing corticosteroid therapy for neurocysticercosis. This patient had also received a one-month course of albendazole prior to treatment with a single dose of ivermectin. Unlike the first case, this patient not only demonstrated persistent RT-PCR positivity despite treatment but also failed to achieve seroreversion or a significant decline in antibody titers (<0.5), suggesting an inadequate therapeutic response.

Of the five patients not completing 12-month follow-up, two died and three were lost to follow-up (LTFU). Deaths occurred on days 28 and 61 following treatment due to prostatic cancer and liver cirrhosis; both were considered unrelated to treatment or strongyloidiasis. Of the three LTFU, two did not return for control at the 3-month visit, and one was lost to follow-up at the 12-month visit, having shown seroreversion at the last clinical appointment (6-month visit).

No patient had any larvae visualization in either stool culture or in concentration techniques during the follow-up visits (Table 2). Seroreversion was achieved in 16/23 participants (69.6%) while 22/23 participants (95.7%) fulfilled criteria for serological cure. *Strongyloides stercoralis* DNA was detected in two patients during the follow-up period: one who fulfilled criteria for serological cure and another who did not.

The proportion of patients achieving clearance of *S. stercoralis* infection, as determined by a reduction in antibody titers (post-/pre-titer < 0.5), remained remarkably consistent between the 6-month and 12-month follow-up evaluations (93.33% and 93.75%, respectively).

**Figure 2:**
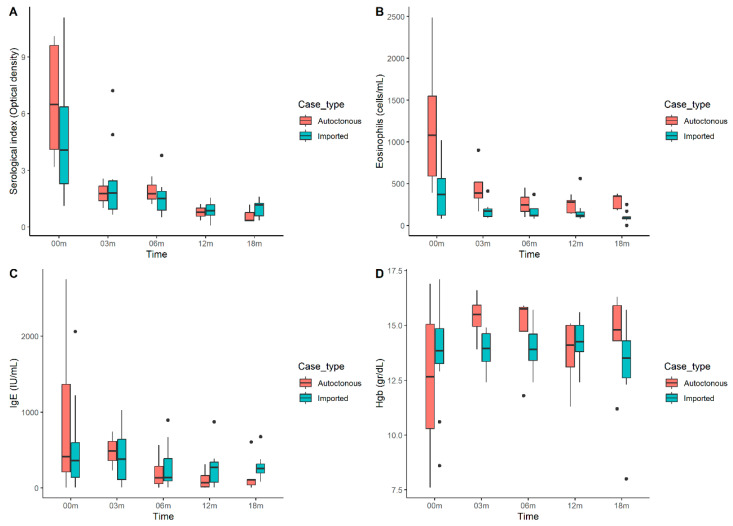
(**A**) Evolution of the serological index over time, (**B**) evolution of the eosinophil count over time, (**C**) evolution of Immunoglobulin E levels over time, and (**D**) evolution of hemoglobin levels over time. (Dots are outliers).

In our series, mild fluctuations in serological titers after treatment were observed. In two cases, a slight increase in serological titers was observed between the 12-month and 18-month follow-up evaluations. Despite this transient elevation, both patients maintained a post-/pre-treatment serological ratio of <0.5 relative to baseline, meeting the established criteria for treatment response. Figure 2 shows serological titers, eosinophil counts, and IgE and hemoglobin levels throughout the visits.

Serological index results, eosinophils, IgE, and Hb over time are represented in Figure 2

## 4. Discussion

This study demonstrates the safety and efficacy of ivermectin therapy for the treatment of strongyloidiasis. Using both serological and molecular methods, 21/28 (75%) patients were cured. This percentage increased to 91% (21/23) when we considered only those patients who completed at least 12 months of follow-up.

Accurate diagnosis of strongyloidiasis and evaluation of treatment response is crucial. The lack of a reliable method for assessing a cure is a significant concern [18], particularly in patients receiving immunosuppressive therapies. These individuals are at increased risk of developing *Strongyloides stercoralis* hyperinfection syndrome, a condition associated with high mortality, which can be mitigated by early diagnosis [5,19].

The intermittent larval excretion and low parasite burden pose challenges for microscopic detection and evaluation of treatment effectiveness [20]. Previous studies [21,22] have shown that analyzing multiple samples is necessary to achieve adequate sensitivity, yet a substantial proportion of infections may still be missed. In chronic strongyloidiasis, the sensitivity of a single stool examination for detecting *S. stercoralis* in asymptomatic individuals is reduced. Notably, traditional parasitological methods failed to detect any cases in our study. This may be attributable to both a lack of experienced parasitologists and the inherently lower sensitivity of these tests in asymptomatic patients with low parasite burdens [9]. We employed the stool concentration system used in routine clinical practice. However, alternative concentration techniques, such as the modified Rugai [23] or Baermann methods, might have yielded better results.

To our knowledge, few studies have evaluated ivermectin efficacy using both serology and stool PCR [10,15,24,25]. In our study, 91% of participants were considered cured after a minimum 12-month follow-up. This cure rate is similar to that reported by Buonfrate et al. [7], who compared single-dose and multiple-dose ivermectin regimens for *Strongyloides stercoralis* infection and found comparable cure rates in both groups. While anti-*S. stercoralis* antibody concentrations typically decrease in successfully treated patients [13,26], our findings, consistent with other reports [7,27], suggest that most serological responses indicative of infection clearance occur within the first 6 months post-treatment. This observed stability between 6 and 12 months suggests that a 6-month follow-up period might be sufficient to assess treatment response in select patient populations, potentially optimizing resource allocation and patient monitoring strategies. Notably, PCR testing in our study identified one treatment failure that would have been missed by serological testing alone.

PCR may have a role in both detecting and monitoring infections, identifying additional cases missed by microscopy or serological testing [24]. In our cohort, PCR proved valuable, identifying one case of parasitological failure undetected by other microbiological methods. Our study observed a PCR negativization rate of 50% (2/4 patients) at 18 months of follow-up, which is higher than the rate described by Repetto et al. [28]. However, it is important to acknowledge that variations in treatment regimens, patient characteristics, and follow-up duration across studies can contribute to differences in PCR negativization rates.

A substantial proportion of patients (53.57%) in this study presented with eosinophilia, supporting its utility as an infection marker, consistent with other studies [19]. While the mean eosinophil count decreased significantly from baseline to the 3- to 6-month follow-up and remained stable at the 12- and 18-month assessments, this decrease was equally observed in both patients who achieved a cure and those who did not. Therefore, unlike the findings of Nuesch et al. [29], eosinophil counts in our study did not reliably predict treatment efficacy, a finding also reported in other trials [7,28].

Elevation of IgE (39.13%) has also been reported in *S. stercoralis* infection at similar rates (38–59%) [18,30]; however, unlike eosinophilia, IgE levels decrease less substantially following treatment and often remain elevated.

Ivermectin, the recommended treatment for strongyloidiasis [21], proved highly effective and well-tolerated in our study, whether administered as a single 200 mg/kg dose or 200 mg/kg/day over two days.

Consistent with previous reports [12,24], our findings suggest that combining PCR and serological testing could maximize sensitivity for detecting *Strongyloides stercoralis* infection and treatment failures. Further research is needed to optimize treatment regimens and establish ideal follow-up strategies, particularly for individuals at increased risk of treatment failure. Also, establishing clear criteria for retreatment would be of great help.

Our study has several limitations. First, the smaller sample size limits our ability to draw definitive conclusions regarding laboratory findings and diagnostic techniques. Second, the presence of both autochthonous and migrant cases in our region, while offering a unique opportunity, also presents potential challenges for interpretation. It is possible that some cases classified as imported may have acquired geohelminth infections within Spain, as some immigrants had resided in the Vega Baja region for extended periods and could have been locally infected. Moreover, other helminthiases that can produce cross-reactions with *Strongyloides* serology were not systematically excluded, although no other helminth infections were found in the coproparasitological analysis performed at each visit. Third, the limited representation of immunosuppressed individuals in our study may limit the generalizability of these results to that population. Fourth, there were significant losses to follow-up, and only a small proportion of participants attended all the scheduled visits. Fifth, the inability to confirm whether patients had visited endemic countries during the follow-up period and the impossibility of excluding an ongoing transmission in the Vega Baja region could potentially contribute to reinfection and influence the assessment of treatment efficacy.

However, our study’s strengths include the use of highly sensitive diagnostic methods to evaluate a cure and a longer follow-up period than many previous studies. Furthermore, the prospective design and specific setting allowed for a direct comparison of different diagnostic methods, enabling a more comprehensive evaluation of their performance in detecting *Strongyloides stercoralis* infection and assessing treatment response.

## 5. Conclusions

Our results demonstrated a high response rate to ivermectin treatment. Serology proved useful for monitoring treatment efficacy; however, PCR identified one additional case of persistent infection, highlighting its complementary role. Notably, eosinophil levels were not found to be a reliable marker of treatment failure. However, further studies with a larger number of patients are needed to confirm these findings and to assess the effectiveness of ivermectin regimens, particularly in immunocompromised patients.

## Figures and Tables

**Table 1:** Patient characteristics of the global cohort and outcomes.

Characteristics	Global Cohort (n = 28)	Autochthonous (n = 8)	Foreign (n = 20)	*p*
Age, years, median (IQR)	54.5(41.0–66.5)	77 (74–81)	49 (37–57)	**<0.001**
	***n* (%)**	***n* (%)**	***n* (%)**	
**Demographic information**				
Gender, women	14 (50)	2 (25)	12 (60)	0.20
**Occupation**				
Farmer	10 (36)	4 (50)	6 (30)	0.50
**Clinical symptoms**				
Pulmonary	2 (7)	1 (12)	1 (5)	0.49
Dermatological	11 (39)	4 (50)	7 (35)	0.67
Gastrointestinal	8 (29)	1 (12)	7 (35)	0.37
**Concomitant diseases**				
Chronic liver disease (no cirrhotic)	1 (4)	1 (12)	0 (0)	0.28
Cirrhotic chronic liver disease	1 (4)	0 (0)	1 (5)	1
Chronic kidney disease without dialysis	3 (11)	3 (38)	0 (0)	0.02
HIV infection	2 (7)	0 (0)	2 (10)	1
Solid neoplasia	1 (4)	1 (12)	0 (0)	0.28
Hematological neoplasia	0 (0)	0 (0)	0 (0)	1
Autoimmune diseases	1 (4)	0 (0)	1 (5)	1
Inflammatory bowel disease	0 (0)	0 (0)	0 (0)	1
Other digestive disease	2 (7)	1 (14)	1 (5)	0.31
Skin disease	2 (7)	1 (12)	1 (5)	0.49
Neurological disease	1 (4)	0 (0)	1 (5)	1
Rheumatologic disease	1 (4)	0 (0)	1 (5)	1
Pneumopathy	2 (7)	1 (12)	1 (5)	0.49
Heart disease	2 (7)	0 (0)	2 (10)	1
**Medication**				
No medication	21 (75)	3 (38)	18 (90)	**0.01**
Laxatives	1 (4)	1 (12)	0 (0)	0.28
Gastroprotector medication	6 (21)	4 (50)	2 (10)	**0.04**
Immunosuppressive drugs	2 (71)	2 (25)	0 (0)	0.07
Corticosteroids	1 (4)	1 (12)	0 (0)	0.28
Chemotherapy	1 (4)	1 (14)	0 (0)	0.25
**Other parasitosis**	12 (43)	0 (0)	12 (60)	**0.01**
Chagas	8 (29)	0 (0)	8 (40)	0.06
Malaria	2 (7)	0 (0)	2 (10)	1
**Laboratory tests (first visit), median (range)**				
Eosinophils (cells/L)	13.6 (12.9–14.9)	1080 (592–1548)	370 (122–560)	**<0.001**
Hb (mg/dL)	510 (205–792)	12.6 (10.3–15.0)	13.8 (13.3–14.8)	0.43
IgE (UI/mL)	371 (148–609)	413 (211–1367)	361 (139–599)	0.68
**Outcomes**	***n* (%)**	***n* (%)**	***n* (%)**	
Death	2 (7)	2 (25)	0 (0)	0.09
LTFU	3 (11)	0 (0)	3 (15)	0.44
Cure	21 (75)	6 (75)	15 (75)	1
Treatment failure	2 (7)	0 (0)	2 (10)	0.6

IQR: interquartile range; Hb: hemoglobin; HIV: human immunodeficiency virus; LTFU: lost to follow-up; **in bold**: statistically significant.

**Table 2:** Clinical manifestations and hematological, serological, parasitological and molecular results during follow-up.

	Visits				
	Baseline (*n* = 28)	Month 3 (*n* = 17)	Month 6 (*n* = 17)	Month 12 (*n* = 17)	Month 18 (*n* = 15)
Symptomatic n/N (%)	15/28 (54%)	2/17 (12%)	2/17 (12%)	1/17 (6%)	0/15 (0%)
Positive serology *n/N* (%)	28/28 (100%)	8/16 (50%)	8/15 (53.3%)	3/16 (18.75%)	5/15 (33.4%)
Seroreversion *n/N* (%)	NA	7/16 (44%)	7/15 (46.7%)	12/16 (75%)	10/15 (66.7)
Post-/pre-titer < 0.5, *n/N* (%)	NA	13/16 (81.2%)	12/15 (80%)	15/16 (93.75%)	15/15 (100%)
Eosinophilia, *n/N* (%)	15/28 (54%)	1/16 (6.3%)	0/13 (0%)	1/15 (6.7%)	0/14 (0%)
Eosinophils, median (IQR)	510 (205–792)	180 (117.5–245.6)	190 (110–194.6)	140 (115–195.3)	135 (92.5–237.5)
Elevated IgE, *n/N* (%)	19/22 (86%)	10/12 (83%)	6/12 (50%)	9/14 (64%)	10/13 (77%)
IgE, median (IQR)	371 (148–609)	381.00 (126.25–416.94)	136.50 (66.85–260.80)	248.00 (25.95–326.50)	205 (95.5–336.50)
Hb, median (IQR)	13.6 (12.9–14.9)	14.45 (13.45–14.85)	14.20 (13.40–14.22)	14.10 (13.50–14.05)	14.05 (12.53–15.03)
Larvae visualization, *n/N* (%)	0/28 (0%)	0/10 (0%)	0/14 (0%)	0/13 (0%)	0/11 (0%)
Stool *Strongyloides* PCR positive, *n/N* (%)	4/16 (25%)	0/9 (0%)	1/13 (7.6%)	2/16 (12.5%)	0/10 (0%)

Hb: hemoglobin; NA: not available; and PCR: polymerase chain reaction.

## Data Availability

All data may be shared and should be requested from the corresponding author.
